# Rethinking perceptual distortions in amblyopia: toward a multi-feature, visual-field-wide perspective

**DOI:** 10.3389/fnins.2026.1763302

**Published:** 2026-04-21

**Authors:** Haneieh Molaei, Reza Farivar

**Affiliations:** 1 McGill Vision Research Unit, Department of Ophthalmology and Visual Sciences, McGill University, Montreal, QC, Canada; 2 Research Institute of McGill University Health Centre, Montreal, QC, Canada

**Keywords:** amblyopia, binocular function, perceptual distortion, perceptual organization, spatial distortions, visual acuity, visual field mapping

## Abstract

Perceptual distortion is a key yet under-recognized feature of amblyopia that extends beyond visual acuity loss and impacts binocular vision. While various forms of distortion have been reported, discrepancies across studies may reflect the narrow focus on single stimuli or distortion types. We argue for a more comprehensive approach to studying distortion—through mapping and multi-feature assessment—as a potentially informative framework for future diagnostic and treatment-related applications, as well as for understanding the neural basis of amblyopia.

## 1 Introduction

Amblyopia is a neurodevelopmental condition that affects approximately 3–5% of children (Clavagnier et al., 2015; Xiao et al., 2015; Mostafaie et al., 2020; McConaghy and McGuirk, 2019; Asare et al., 2023), making it one of the most prevalent eye diseases in Canada and worldwide (Asare et al., 2023; Drover et al., 2008). This condition involves various deficits, including reduced visual acuity (VA), low contrast sensitivity, weakened binocular functionality, and visual distortion. Although more than 200 years have passed since the first scientific publications on amblyopia, visual distortions in amblyopia was first identified in the 1950s (Pugh, 1958) and remains a significant area of research due to its impact on both visual perception and function. In this review, we provide a comprehensive examination of the evidence regarding the presence, distribution, and visual field (VF) prevalence of distortions in amblyopia.

In this review, we use the term perceptual distortion as an umbrella concept to describe systematic alterations in the perceived appearance or spatial properties of visual stimuli, rather than simple losses of visibility or resolution. Perceptual distortions can manifest in multiple ways, including positional displacement (mislocalization of stimulus elements), orientation distortions, changes in perceived spatial frequency or scale, and more complex alterations in perceived form or structure. These phenomena are treated here as distinct but related manifestations of distorted visual representation, and together constitute what we refer to as perceptual distortion in amblyopia.

This article is intended as a Perspective rather than a comprehensive review of all visual deficits in amblyopia. We focus specifically on perceptual distortions, drawing on behavioral studies to highlight their prevalence and variability, and to motivate a multi-feature, visual-field–wide approach to their measurement and interpretation. Throughout the manuscript, we distinguish between conclusions that are supported by existing evidence and ideas that represent proposed or future directions.

Section 1 focuses on why perceptual distortion represents a central aspect of amblyopia that is not captured by visual acuity alone, drawing on behavioral evidence and links to functional outcomes, including binocular vision. Section 2 then addresses why estimates of distortion vary across studies, considering differences in stimulus type, subject characteristics, measurement approach, and stimulus size or spacing, and highlights what conclusions remain consistent across this variability. Section 3 broadens the discussion beyond the fovea to the visual field more generally, emphasizing evidence for extrafoveal distortion and the value of mapping spatial patterns of distortion. Finally, Section 4 presents a conceptual, hypothesis-driven perspective on how behavioral distortion maps may relate to known principles of cortical organization and outlines directions for future work.

## 2 The importance of distortions in amblyopia

In this section, we summarize evidence that perceptual distortions are common in amblyopia and argue that it reflects more than reduced acuity, motivating the need to measure distortions directly.

### 2.1 Prevalence of distortions

Previous studies have explored various forms of visual distortion in amblyopia, including letter deformation (Pugh, 1958; Pugh, 1962), spatial displacement (Iftime et al., 2007; Mansouri et al., 2009; Sireteanu et al., 1993; Sireteanu et al., 2008; Sireteanu et al., 2008; Fronius and Sireteanu, 1989; Piano et al., 2016; Piano et al., 2015), orientation errors (Barrett et al., 2003; Sharma et al., 1999; Hess and Malin, 2003; Skottun et al., 1986; Rentschler and Hilz, 1979), altered SF perception (Barrett et al., 2003; Skottun et al., 1986; Hess et al., 1978), and missing visual information in gratings (Iftime et al., 2007; Sireteanu et al., 1993; Barrett et al., 2003; Hess et al., 1978).

For example, Barrett et al. (2003) and Piano et al. (2015) found that more than two-thirds of amblyopic participants perceived distortion when tested with a specific stimulus and only when viewing with the AE. While this figure is substantial, it likely underestimates the overall prevalence of distortion in amblyopia, as most studies examine only one distortion type using a single stimulus. Our recent study (Molaei et al., 2025a) supports this, showing that the prevalence of distortion varies markedly across stimulus types (e.g., high for SF, moderate for position, low for orientation), meaning that some amblyopes show distortion for one stimulus but not another. To accurately assess how widespread visual distortions truly are, large-scale studies are needed that evaluate multiple distortion types within the same cohort.

### 2.2 Perceptual distortion vs. VA and binocular vision

VA measurement has long served as the primary indicator of visual function in amblyopia, used to diagnose the condition and monitor improvements over time (McConaghy and McGuirk, 2019; Wallace et al., 2018). However, in the presence of perceptual distortions, VA measurements may not capture the actual quality or accuracy of the visual percept. VA remains an important clinical measure, but on its own it cannot diagnose amblyopia; amblyopia is a diagnosis of exclusion, and VA reflects only one aspect of visual function.

Although acuity charts are highly standardized, they still reduce vision to a recognition score and may miss systematic changes in the perceived form of the stimulus.

**Figure 1:**
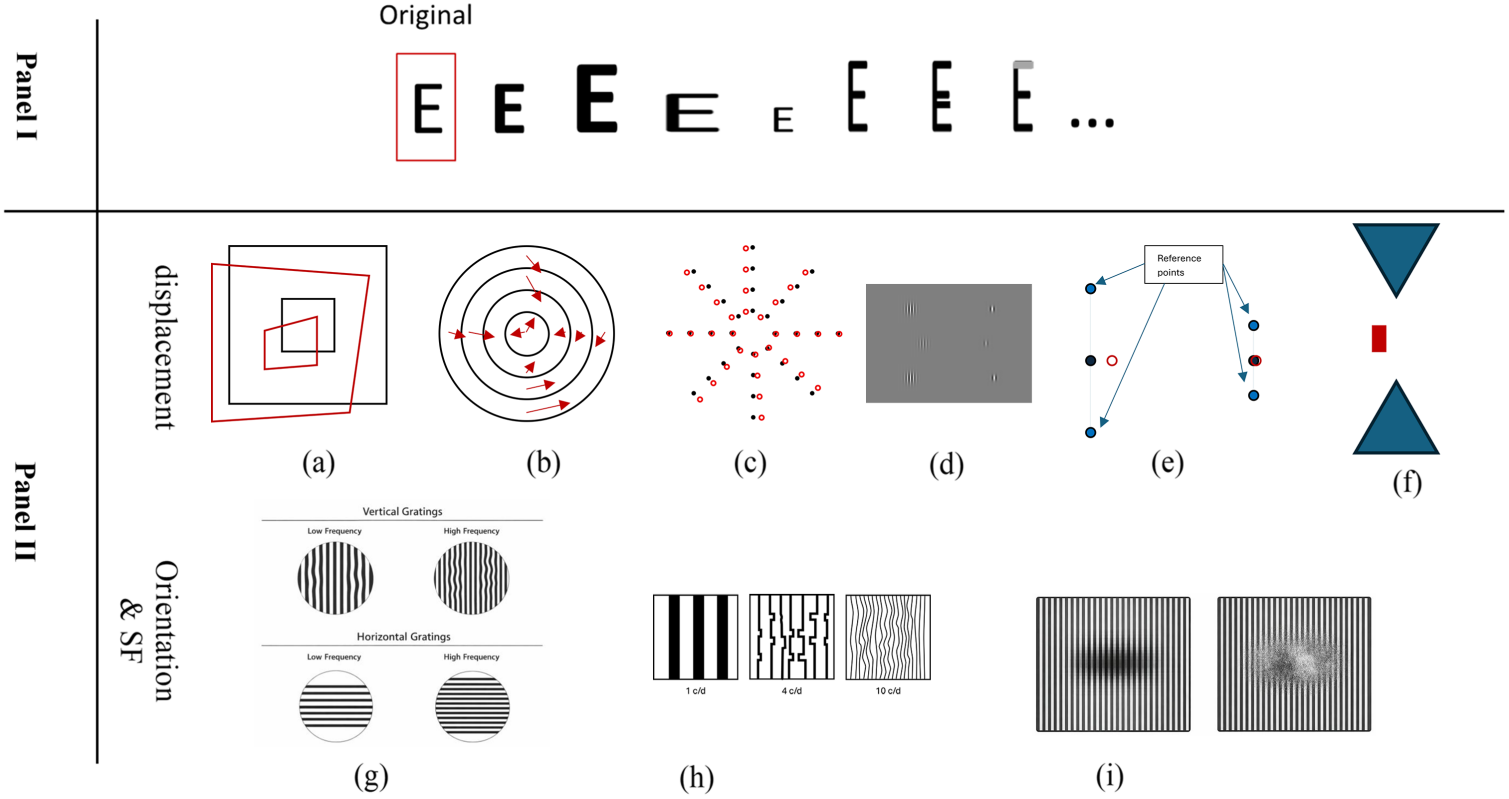
(Panel I) Various possible perceptions of the letter “E.” Moving from left to right: Original, bold, enlarged and bold, horizontally expanded and vertically bold, smaller, vertically expanded, vertically expanded with additional horizontal lines, vertically expanded and partially faded. Many other perceptual variations are possible. (Panel II) Examples of visual distortions perceived with AE. The displacement distortion using groups of blobs in the form of rectangular **(a)** and circular patterns **(b,c)**, using Gabors **(d)**, single blobs **(e)**, and hour-glass shaped fiducial target **(f)** in the form of vertical alignment. The orientation distortions using gratings in horizontal and vertical directions **(g)**. The increment of distortion in higher SF using gratings **(h,i)** adapted from Sireteanu et al. (2008), Fronius and Sireteanu (1989), Piano et al. (2015), Barrett et al. (2003), Bedell and Flom (1981), Mansouri et al. (2009), Hess and Field (1993) and Hess et al. (1978).

These contrasting approaches raise the question of what VA measurements actually capture. VA can be assessed through either resolution tasks—such as the Landolt C, which measures the minimum angle of resolution (MAR)—or recognition tasks that require identifying letters or pictures. Recognition-based charts, including ETDRS, rely on identifying familiar optotypes and therefore may not be sensitive to subtle changes in shape or structure. The illustrative example in Figure 1 panel I, shows how an AE could perceive small form-level distortions in a letter “E” while still identifying it correctly, leaving such distortions undetected in a standard VA score. Thus, while VA remains an essential clinical measure, it does not necessarily reflect the full quality of the visual percept or the presence of perceptual distortions.

Interestingly, several studies have reported no clear correlation between VA loss and key visual deficits in amblyopia, including visual distortion (Pugh, 1958; Sireteanu et al., 2008; Fronius and Sireteanu, 1989; Piano et al., 2016), binocular function (Webber et al., 2020; Shiraishi et al., 2023; Hozumi et al., 2023; Zhao et al., 2017), and suppression (Shiraishi et al., 2023; Hozumi et al., 2023; Chen et al., 2020; Webber et al., 2020; though some studies do report a correlation; Hess et al., 2014; O'Connor et al., 2010; Hess et al., 2011; Lv et al., 2024; Birch, 2013). The severity of visual distortions may not align with the level of VA loss, even after improvements in VA (Pugh, 1958; Sireteanu et al., 2008; Piano et al., 2016). Other studies (Sireteanu et al., 2008; Fronius and Sireteanu, 1989; Piano et al., 2016; Bedell and Flom, 1981) have artificially induced further VA loss to explore potential links between visual distortions in the AE and interocular VA difference, yet no correlation has been detected. We can therefore conclude that VA measurements do not reveal all critical aspects of amblyopia and should not be the sole parameter used for assessment. Although VA remains an essential clinical measure, many aspects of daily visual performance—such as depth perception, binocular alignment, fusion stability, and spatial accuracy—depend more directly on binocular function and the integrity of the percept itself. Distortion, suppression, and binocular imbalance can disrupt these functions even when VA is normal or has improved, making them more critical for understanding how amblyopia affects functional vision. For this reason, VA should be interpreted alongside these additional measures rather than used as the sole indicator of visual status.

In contrast to VA, monocular visual distortions have been implicated in binocular difficulties, including abnormalities in fusion, stereoacuity, and suppression in at least a subset of amblyopes (Piano et al., 2015; Piano et al., 2016; Webber, 2018; Wright, 2003; Zagui, 2019). These binocular functions support everyday depth perception and spatial coordination, and many amblyopes show reductions in one or more of them (Hess et al., 2011; Birch, 2013; Chadnova et al., 2017; Hess and Thompson, 2015; Hess, 1996; Gaier and Hunter, 2017; McKee et al., 2003). Because approximately 70% of amblyopes report monocular distortions (Piano et al., 2015; Barrett et al., 2003), such distortions may contribute to mismatches between the two eyes’ images that make binocular integration more difficult. Although the available evidence is largely correlational and relationships are not always consistent across studies, this association suggests that distortions should be considered alongside VA when evaluating binocular function in amblyopia.

Importantly, the studies cited above primarily report associations between perceptual distortion and other visual deficits in amblyopia, such as binocular dysfunction, suppression, and reduced stereoacuity. These findings are largely correlational and do not directly address underlying mechanisms or establish causal relationships between distortion and other impairments.

Accordingly, existing evidence does not allow perceptual distortion to be interpreted solely as a downstream consequence of other amblyopia-related impairments, nor as a fully independent deficit, but rather suggests that distortion may reflect a partially dissociable perceptual alteration that interacts with other visual deficits.

### 2.3 Research values of distortion

While the studies of distortions discussed above jointly support the importance of studying visual distortions as a compliment to VA in understanding amblyopia deficit, studies of visual distortion merit greater consistency, rigour and power. Reported estimates of distortion nevertheless vary substantially across studies, reflecting differences in stimulus properties, participant characteristics, and measurement approaches.

The dependence of distortion on stimulus properties and measurement approach is discussed in detail in Section 2, where we summarize what can (and cannot) be generalized across studies.

While these findings primarily establish the research value of studying perceptual distortions, they also motivate consideration of their potential clinical relevance.

Therefore, given that (Clavagnier et al., 2015) perceptual distortions in the AE are *associated with* disruptions in binocular functionality and fusion (Xiao et al., 2015), are often overlooked when evaluating VA loss, and (Mostafaie et al., 2020) are reported by a substantial proportion of amblyopes (approximately 70%), we argue that visual distortions may have clinical relevance and warrant continued investigation.

A key challenge, addressed in the next section, is to determine which aspects of distortion are robust across tasks and which are stimulus- or method-specific.

### 2.4 Distortion estimates depend on stimulus type, subjects, extracting method, and stimulus size

Having established why distortion matters, we next address why the literature can appear inconsistent—reported distortions depend strongly on stimulus properties, subject characteristics, and the methods used to measure them.

### 2.5 Stimulus type and distortion

Each study on distortion in amblyopia has typically focused on a single distortion type—such as deformation, displacement, misorientation, or SF changes—using a stimulus tailored to reveal that specific form. These include gratings/Gabors (Barrett et al., 2003; Sharma et al., 1999; Hess and Malin, 2003; Vandenbussche et al., 1986; Yap et al., 2020; Bäumer and Sireteanu, 2006; Hess and Field, 1993; Kirsch and Kunde, 2023; Sireteanu et al., 2008; Barrett et al., 2003; Hess and Malin, 2003; Hess et al., 1978; Dallala et al., 2010; Kwon et al., 2015), blobs (Mansouri et al., 2009; Fronius and Sireteanu, 1989; Piano et al., 2016; Piano et al., 2015), letters (Pugh, 1958; Pugh, 1962), or hourglass-shaped fiducial targets (Bedell and Flom, 1981; Bedell et al., 1985) presented to the AE. The variation in both stimulus type and distortion type across studies highlights that different distortions may be more easily revealed depending on the specific visual input. This suggests that a single subject might experience multiple types of distortion (Molaei et al., 2025a), but these may only emerge under particular stimulus conditions. Therefore, to understand the full scope of perceptual distortion in amblyopia, it is important to examine how different stimuli can uncover distinct distortion profiles.

Pugh (1958) was the first to investigate visual distortion in amblyopia using letter-based stimuli. She employed lines of letters from a Snellen chart, and participants described perceiving the letters as jagged, dragged, smudged, or faded—rather than clearly defined—indicating distortions in letter shape perception. This foundational observation led to subsequent research that sought to explore the phenomenon further using simpler and more controlled visual stimuli.

Spatial displacement or position distortion in the AE has been studied using various stimuli and methods, including (see Figure 1 panel II, a–f; Clavagnier et al., 2015). Arranging blobs in circular (Iftime et al., 2007; Sireteanu et al., 1993; Sireteanu et al., 2008; Sireteanu et al., 2008; Mansouri et al., 2009) or rectangular (Piano et al., 2015; Piano et al., 2016) patterns across the VF (from 5° to 30°; Xiao et al., 2015). Aligning a Gabor/grating with two other Gabors/gratings in veridical meridian (Hess and Malin, 2003; Hess and Field, 1993; Hess and Field, 1994; Mostafaie et al., 2020). The same alignment task using blobs/lights (Fronius and Sireteanu, 1989) instead of Gabor/grating; and (McConaghy and McGuirk, 2019). The same alignment task with an hourglass-shaped fiducial target (Bedell and Flom, 1981; Bedell et al., 1985). Each of these methods aimed to manifest the displacement distortion in AE.

Orientation distortion, as another type of visual distortion, has also been assessed in several studies. The direction and SF of Gabor or grating patterns play a role in orientation distortion in the AE. Studies (Barrett et al., 2003; Rohr et al., 2022) suggest that vertical orientations are perceived less veridically than horizontal orientations. Even the location of the stimuli matters: horizontal orientations are perceived more accurately on the horizontal meridian than on the vertical meridian (Kirsch and Kunde, 2023). Additionally, higher SFs lead to greater perceived distortion of the stimuli (Sharma et al., 1999; Skottun et al., 1986; Levi, 2013; Hess et al., 1978; Demanins et al., 1999a, 1999b; Mansouri et al., 2007; see Figure 1 panel II g).

Distortions related to changes in SF have been primarily examined with Gabors/Gratings and, in rare cases, with letters as stimuli (Kwon et al., 2015). As expected, the higher the SF, the more challenging it becomes for the AE to perceive it accurately (Sharma et al., 1999; Skottun et al., 1986; Barrett et al., 2003; Levi, 2013; Demanins et al., 1999a, 1999b; Mansouri et al., 2007; see Figure 1 panel II, h–j). A question arises: is the distortion observed when testing high-SF Gabors or gratings due to the high SF itself or the orientation? It is likely due to high SF, as it is well-documented that the AE struggles significantly with higher SFs (Kwon et al., 2015; Zhou et al., 2015; Mao et al., 2020; Wiecek et al., 2024). This might suggest that using low SFs to test for orientation distortion would likely yield results more specific to orientation distortion rather than SF distortion. The same applies to SF distortion, where horizontal Gabors/gratings may be recommended over other orientations to ensure results reflect the effects of SF alone.

This underscores the importance of selecting appropriate stimuli to accurately extract each type of distortion. Using an inappropriate stimulus may yield misleading results, as could happen when assessing orientation distortions with high-SF Gabors or gratings. Similarly, using Gabors/gratings to extract displacement distortions may not be ideal, as SF perception could distort positional accuracy.

Would using more complex stimuli engage more types of distortions in AE perception? When real images (Sireteanu et al., 2008; Sireteanu et al., 1993; Hess et al., 1990) and letters (Pugh, 1958; Pugh, 1962) are presented to the AE, the quality and quantity of distortions may differ significantly from simpler elements like blobs or Gabors/gratings. It remains unclear whether distortions in real image perception are a combination of existing distortion types or represent a novel and separate type of distortion (Sireteanu et al., 2008). Sireteanu et al. (1993) found that when a displacement distortion patterns—originally extracted from a circular blob pattern—was applied to reconstructed images, the resulting distortions were excessive for the AE to perceive accurately. While some similarities were noted in the distortion of lines and edges, the AE perceived less distortion than the generated pattern suggested, possibly due to compensatory mechanisms that improve AE perception beyond the expected distortion level. Thus, extracting distortion patterns for realistic images is more complex than assessing distortions in simple stimuli.

### 2.6 Interaction of subject selection and observed distortions

Selection of participants is important, whether in studies with control participants (Himmelberg et al., 2020; Morsi et al., 2024) or with amblyopia participants (Chung et al., 2006). Amblyopia is a disease with a spectrum of intensity—from mild to severe—and varying etiologies (strabismic, anisometropic, or mixed), each with unique features and potential deficits (Duman et al., 2018; Wiecek et al., 2024; Nasr et al., 2024). Therefore, participant factors should not be overlooked. Research has shown that several key deficits differ in magnitude or pattern between strabismic and anisometropic amblyopia, including contrast sensitivity, peripheral binocular imbalance, ocular-dominance organization, and structural/functional brain alterations in visual pathways (Wiecek et al., 2024; Nasr et al., 2024; Naheed et al., 2024; Wang et al., 2023). One possible explanation for contradictory findings across studies, despite identical methods, stimuli, and procedures, is the substantial variation among amblyopic subjects—including differences in amblyopia subtype and severity of visual deficits (Birch, 2013; Barrett et al., 2004; Simmers and Bex, 2004). This suggests that if past studies had been conducted on the same amblyopic group, the results would likely be more trustworthy and reliable, as the subjects would have shared similar visual and physical conditions. This is especially true if the study were looking for possible correlations in clinical features or deficits in amblyopia.

Because the influence of subject variability has not been systematically examined, it is unclear how much this factor may have shaped previous findings—whether by masking a true effect, exaggerating it, or contributing to the contradictory results reported across studies.

### 2.7 Method and distortion

The primary methodologies used to capture visual distortions in amblyopia include: (1) asking subjects to describe or draw what they see (Pugh, 1958; Iftime et al., 2007; Sireteanu et al., 1993; Sireteanu et al., 2008; Barrett et al., 2003; Hess et al., 1978; Ahmad et al., 2023), (2) adjustment-based procedures in which the fellow eye’s (FE) blobs or cursor are manipulated to match the AE’s percept (Sireteanu et al., 1993; Sireteanu et al., 2008; Sireteanu et al., 2008; Piano et al., 2015; Mansouri et al., 2009), and (3) using a two-alternative forced-choice (2AFC) method where subjects press keys to indicate the position of vertical lines (Fronius and Sireteanu, 1989; Bedell and Flom, 1981; Bedell et al., 1985). If two studies were to use identical stimuli and identical observers but different measurement methods, it remains uncertain whether they would report distortions of the same magnitude or even the same qualitative structure. Methodological choices—including the response format, degree of control given to the observer, task duration, and experimental environment—can influence how precisely distortions are captured, even if the overarching interpretation (i.e., that distortions exist) remains unchanged. Indeed, even moving from an online platform to a lab-based setting can alter the robustness or statistical significance of results while preserving the general pattern (Kirsch and Kunde, 2023).

Each method comes with its own strengths and limitations. For example, 2AFC procedures are criterion-independent and can be highly reliable and efficient when implemented with adaptive staircases; however, they constrain the observer to selecting between only two alternatives per trial. Because many amblyopic distortions are multidimensional—often combining blur, positional warping, spatial-frequency changes, or wavelike patterns—it is possible that a limited 2AFC response space may not fully represent the richness or complexity of these percepts. Representing all combinations of distortion parameters in a forced-choice format could require very large stimulus sets or extremely long experiments. Shorter procedures, on the other hand, necessarily restrict the number and range of distortions that can be sampled, increasing the risk that certain aspects of the percept may be missed or under-sampled.

Adjustment-based approaches offer complementary advantages: they provide continuous control over several stimulus parameters at once, which may allow observers to approximate complex percepts involving multiple interacting distortion types. However, adjustment methods are also more subjective—they rely heavily on the observer’s interpretation of the available controls and their ability to manipulate several parameters simultaneously—which introduces its own variability.

These differences may be further amplified by the well-documented fatigue effects in amblyopia. When the AE is fatigued, its efficiency drops markedly during sustained psychophysical tasks (Pons et al., 2019; Polat et al., 2004; Hou and Nicholas, 2022; Li et al., 2008). Faster methods may therefore yield more stable or reliable estimates under fatigue, whereas longer or more cognitively demanding ones may introduce variability. Importantly, this does not imply that any particular method is superior; rather, each method may be differentially sensitive to fatigability, complexity of percept, or stimulus type.

Overall, these considerations highlight that drawing, adjustment procedures, 2AFC, and other emerging methods each carry distinct advantages and limitations. No study has yet systematically compared these methods using the same stimuli and observers, so their relative sensitivity, reliability, or suitability for different distortion types remains unknown. Clarifying these methodological distinctions represents an important direction for future research.

### 2.8 Stimulus size and distortion

It has long been known that higher target density increases perceptual interference, as reflected by the classic “crowding effect” (Pugh, 1958; Mathew et al., 2011; Bonneh et al., 2007). This effect can also occur with larger stimuli, such as a large Gabor or gratings, compared to smaller ones. Specifically, distortions captured from small stimuli (e.g., covering 1° of the VF) are likely to differ in intensity and even quality from those captured from large stimuli (e.g., 10° or more of the VF), despite identical methods, subjects, and stimulus types—which suggests that the spatial extent of a stimulus can influence how visual information is encoded. For instance, Vandenbussche et al. (1986) tested orientation discrimination using bars of different lengths and found that shorter bars made the task substantially more difficult for amblyopes, who showed larger orientation discrimination thresholds than controls. Kirsch and Kunde (2023) observed that perception accuracy varies with stimulus location in the VF, with stimuli on the horizontal meridian perceived more accurately than those on the vertical meridian, implying that eccentricity and meridional biases shape perceptual responses. Or, Hussain and McGraw (2022) suggested that positional distortion in the VF depends not only on eccentricity but also on the distance between visual elements (i.e., proximity effects sometimes associated with crowding). They emphasized that these proximity-related interference effects can appear across the VF, not only centrally, particularly at small separations.

While none of these studies directly compared distortion measurements obtained from *different stimulus sizes*, together they highlight mechanisms—eccentricity, spatial integration, crowding, spatial uncertainty—that could plausibly cause small and large stimuli to reveal different aspects of distortion, even under identical testing conditions.

Given these mechanistic observations, one possibility is that smaller, localized stimuli may better isolate “local” distortion characteristics—because they occupy a limited portion of the VF that is less affected by eccentricity-driven spatial imprecision and less susceptible to crowding-related interactions. Conversely, larger stimuli might engage more global perceptual processes, potentially revealing broader, more integrated distortion patterns across space. However, it is important to emphasize that this remains speculative, as no study has yet systematically evaluated how stimulus size alters the measured magnitude or nature of perceptual distortions.

If the research goal is to assess distortions across multiple locations within the VF, using small, localized stimuli (e.g., ≤ 0.5°–1°) at different positions may be useful, as these reduce confounding effects of eccentricity and inter-element spacing. In such designs, stimulus size can be scaled with eccentricity (e.g., using cortical magnification factors) to maintain comparable visibility and processing demands across the VF. For questions targeting overall or “global” perceptual organization—such as distortions present in complex images—larger stimuli could be informative, though empirical testing is needed to determine whether these expectations hold.

Overall, variability across stimulus types, subject selection, measurement methods, and stimulus parameters reflect the multi-factorial nature of perceptual distortion across studies. A consistent conclusion is that different experimental choices can emphasize different aspects of distortion, making direct comparisons difficult when multiple parameters vary simultaneously. This highlights the importance of experimental designs that systematically control task structure, stimulus size, and participant group while varying one factor at a time, allowing the specific contribution of that factor to perceptual distortion to be isolated. Recent studies adopting such controlled approaches—manipulating stimulus class or letter form while holding other parameters constant—illustrate how isolating individual parameters can yield more interpretable and internally consistent estimates of perceptual distortion (Molaei et al., 2025a; Molaei et al., 2025b).

## 3 Mappable spatial distortion patterns across the VF

We then extend this framework beyond the fovea and argue that distortions should be considered across the visual field, where normal peripheral imprecision and amblyopia-related abnormalities can interact and where spatial mapping becomes especially informative.

### 3.1 Distortion beyond the fovea

The current dominant view is that the fovea is the main region of vision where AE deficits are most pronounced (Blakemore and Vital-Durand, 1986; Barnes et al., 2001; Hess, 2001; Barrett et al., 2004; Pardhan and Whitaker, 2000) and amblyopia is often described as a primarily “foveal” disorder. However, it is important to note that the periphery of the VF is *not veridical even in normal observers*; positional uncertainty, spatial warping, and crowding increase with eccentricity in the normal visual system. In normally sighted observers, these peripheral imprecisions are systematic, scale predictably with eccentricity, and are largely symmetric across the visual field. Given this, it is reasonable to expect that AE—which already show reduced precision—would exhibit at least the same, and often greater, peripheral distortions. Indeed, large stimuli extending into the periphery are perceived more distorted in the AE than normal periphery (Sireteanu et al., 2008; Barrett et al., 2003; Skottun et al., 1986; Hess et al., 1978; Hess and Field, 1993; Lagreze and Sireteanu, 1991). In contrast, peripheral distortions in amblyopia are typically larger than expected based on eccentricity alone and often show spatial irregularities that exceed the level of imprecision observed in normal peripheral vision under matched viewing conditions.

For example, Sireteanu et al. (1993) reported that their mapping procedure could not capture foveal distortion because their stimuli began beyond 1° of eccentricity, resulting in a displacement map restricted to extrafoveal locations—but critically, the magnitude of extrafoveal distortion in amblyopia was far greater than that of normally sighted controls tested under identical conditions. Hussain and McGraw (2022) further demonstrated that positional judgments are influenced not only by eccentricity but also by the spacing between elements. This suggests that greater distortion observed in the periphery may reflect the combined influence of increased eccentricity and stronger proximity-related interactions (often described within the crowding literature). Importantly, such interactions depend primarily on the spacing between elements rather than on eccentricity alone, meaning they can occur in both foveal and peripheral regions.

Neural evidence for foveal abnormalities—such as reduced cortical magnification in the amblyopic fovea (Hussain et al., 2015; Jigo et al., 2023)—does not negate the presence of peripheral distortion. Although one study reported normal CM in the fovea of the AE (Clavagnier et al., 2015), the observation of reduced CM does not necessarily imply that peripheral distortions are absent. Importantly, the mechanisms underlying distortion have not been definitively linked to the under-sampling hypothesis (Hess et al., 1978; Hess et al., 1990; Williams, 1985; Thibos and Bradley, 1995; Hess and Anderson, 1993; Thibos and Bradley, 1993); several studies instead support the scrambling hypothesis as a plausible alternative (Fronius and Sireteanu, 1989; Hess et al., 1990; Hess, 1982; Field and Hess, 1996; Zhu et al., 2024). Moreover, Kalpadakis-Smith et al. (2022) showed that the mechanisms underlying foveal distortion in amblyopia resemble those producing peripheral distortion in normal vision, highlighting overlapping processes rather than mutually exclusive explanations.

Finally, multiple studies mapping displacement distortions across wide eccentricities (up to 30°) in amblyopia (Iftime et al., 2007; Sireteanu et al., 1993; Sireteanu et al., 2008; Sireteanu et al., 2008; Piano et al., 2015; Mansouri et al., 2009; Hess et al., 1978) consistently show that peripheral distortion is present and is typically larger than the level of distortion observed in the normal periphery. Together, these behavioral and neural findings demonstrate that distortions in amblyopia are not confined to the fovea but extend well into the periphery, where they tend to be exaggerated relative to normal peripheral vision.

### 3.2 From mapping in amblyopia to VF performance in controls

Mapping spatial distortion in the AE not only demonstrates the presence of distortions beyond the fovea but also shows that this distortion is mappable in amblyopia. Previous studies have primarily mapped displacement across the VF of the AE or FE using dots or bulbs at various locations in circular (Iftime et al., 2007; Sireteanu et al., 1993; Sireteanu et al., 2008; Sireteanu et al., 2008; Mansouri et al., 2009; Sireteanu et al., 2007) or rectangular (Piano et al., 2015; Piano et al., 2016) patterns. Li et al. (2017) mapped suppression across the VF in amblyopia by adjusting contrast of a patch in their FE to match the contrast perceived in the corresponding patch in their AE. However, no other studies have attempted to map other types of distortion, such as SF or orientation perception, or more complex tasks like face recognition across the VF in amblyopia.

**Figure 2:**
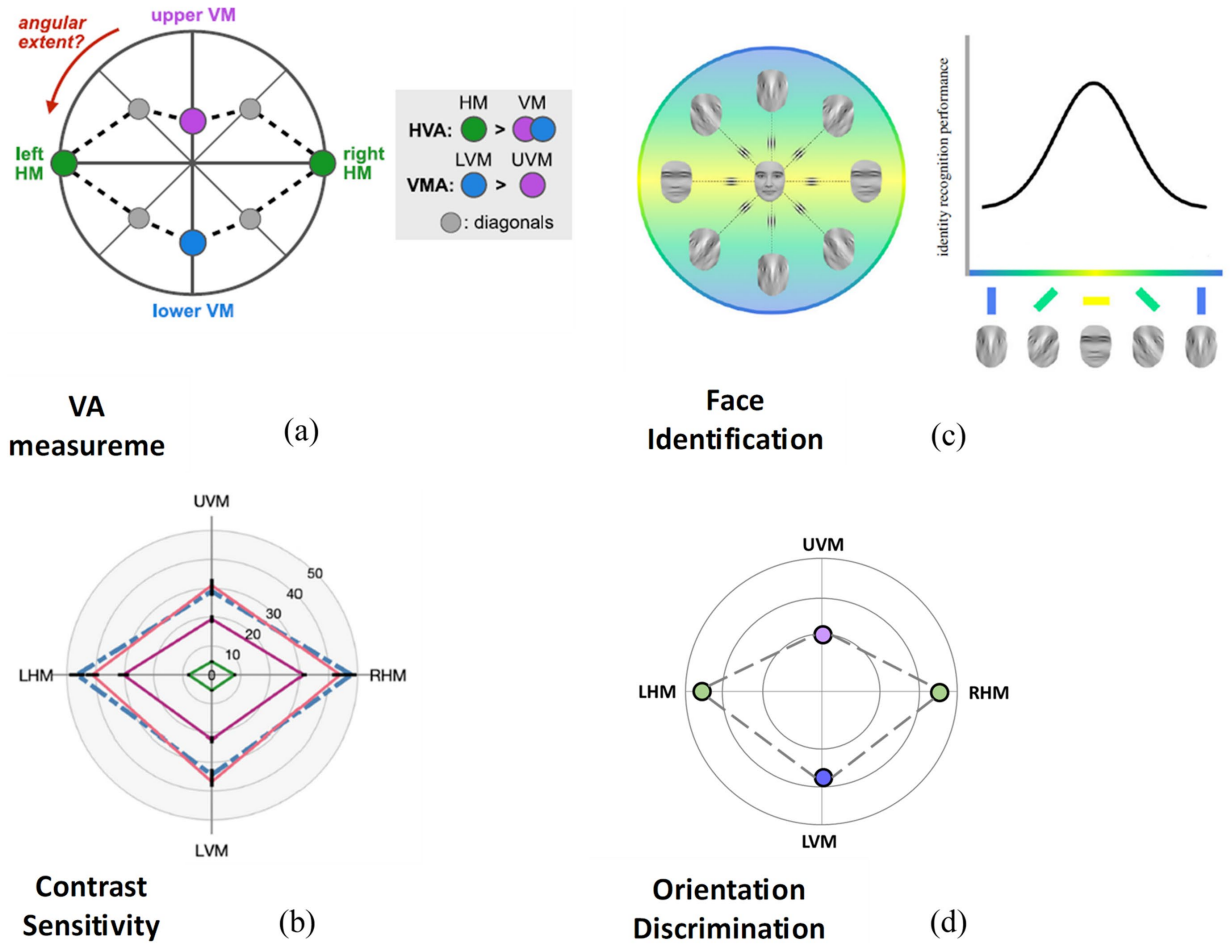
Examples of visual performance fields in normal vision. Visual performance decreases from its highest level along the horizontal meridian (with symmetric performance between the left and right sides) to lower levels on the lower vertical meridian and then the upper vertical meridian. **(a)** Shows VA performance measured across all four major meridians (0°, 45°, 90°, 135°; Barbot et al., 2021). **(b)** Displays contrast sensitivity performance across the two main meridians (0°, 90°; Himmelberg et al., 2020). **(c)** Presents face identification performance across the four major meridians (0°, 45°, 90°, 135°; Roux-Sibilon et al., 2023). **(d)** Illustrates orientation discrimination performance across the two main meridians (0°, 90°; Carrasco et al., 2022).

In contrast, mapping various perceptual features has been extensively studied in individuals with normal vision, including SF (Kirsch and Kunde, 2023), orientation perception (Kirsch and Kunde, 2023; Carrasco et al., 2022), VA (Tsai et al., 2024; Barbot et al., 2021), contrast sensitivity (Jigo et al., 2023; Himmelberg et al., 2022), and face recognition (Morsi et al., 2024; Roux-Sibilon et al., 2023). These studies reveal a largely systematic pattern: visual performance is generally better along the horizontal meridian than the vertical, and on the vertical meridian, performance is better in the lower than the upper field, while performance on the horizontal sides is symmetric (see Figure 2).

If we consider this asymmetric yet systematic pattern of VF performance as a baseline for the visual system, we can investigate the unique patterns of these features in amblyopia. Such patterns could serve as diagnostic tools, markers for tracking improvement, or criteria for assessing treatment outcomes. At present, however, most evidence linking perceptual distortion measures to diagnosis or treatment outcomes is correlational, and their clinical utility remains to be established through longitudinal and interventional studies. They may also provide insights into the behavioral and neural characteristics of amblyopia, revealing unknown aspects of the disease. By mapping these perceptual features and comparing them with normal vision maps, we could explore the neural basis of amblyopia and gain a more complete understanding of the condition. Comparing these maps to those of individuals with normal vision could reveal which aspects of amblyopia resemble typical vision and which differ across various areas of the VF. Are there any similarities? If so, to what extent?

### 3.3 Consistency and uniqueness of distortion

Because distortions in amblyopia are stable over time (Sireteanu et al., 1993; Sireteanu et al., 2008; Piano et al., 2015; Barrett et al., 2003) and each individual has a unique reliable distortion map (Sireteanu et al., 1993; Sireteanu et al., 2008; Piano et al., 2015), mapping offers a novel window into this condition, its causes, effects, and visual cortex activity. This approach provides a more holistic understanding than testing a single stimulus type in specific VF locations, as each map can serve as a ‘fingerprint’ unique to each individual with amblyopia.

## 4 Behavioral-neural bridge via distortion maps

In this section, we outline a hypothesis-driven but explicitly speculative perspective on how behavioral distortion maps might relate to principles of cortical organization. The goal is to provide a conceptual framework that generates testable predictions, rather than to propose established neural mechanisms.

The behavioral dimensions quantified in distortion mapping—spatial position, spatial frequency, orientation, and eye-of-origin—correspond to feature dimensions that are systematically represented in retinotopically organized visual cortex (Dumoulin and Wandell, 2008; Yacoub et al., 2007; LeVay et al., 1985; Yacoub et al., 2008; Hubel and Wiesel, 1998; Broderick et al., 2022; Movshon et al., 1978). In normally sighted observers, these features are encoded through overlapping and interacting maps in early visual areas, particularly V1–V3, where retinotopy, ocular dominance, orientation, and spatial frequency are jointly organized within local cortical neighborhoods (Dumoulin and Wandell, 2008; Yacoub et al., 2008; Goulet and Farivar, 2025; Kim et al., 1999; Blasdel et al., 1995; Blasdel and Salama, 1986; Hubeland and Wiesel, 1977; Lygo, 2019; Clavagnier et al., 2015; Li et al., 2007a). This feature-based organization reflects a fundamental principle of early visual processing, in which multiple visual attributes are encoded simultaneously at each retinotopic location (Nauhaus et al., 2012; Obermayer and Blasdel, 1993; Tootell et al., 1988).

Extensive evidence from animal models demonstrates that amblyopia disrupts this organization across multiple feature dimensions, including degraded retinotopic precision (Swindale and Mitchell, 1994; Yinon, 1976; Baker et al., 1974; Yinon, 1975; Berman and Murphy, 1981; Chino et al., 1983; Blasdel and Campbell, 2001; Yu et al., 2005), shifts in ocular dominance balance (Horton et al., 1997; Kiorpes et al., 1998; Horton and Stryker, 1993; Crawford and Harwerth, 2004; Smith et al., 1997; Movshon et al., 1987; Shooner et al., 2015; Hendrickson et al., 1987), and impaired spatial frequency tuning of amblyopic-eye driven neurons (Kiorpes et al., 1998; Movshon et al., 1987; Shooner et al., 2015). Human neuroimaging studies reveal closely parallel findings, showing enlarged and disordered population receptive fields (Clavagnier et al., 2015; Szinte et al., 2024), increased interocular positional scatter (Dumoulin and Wandell, 2008; Lygo, 2019; Clavagnier et al., 2015; Li et al., 2007a; Chang et al., 2025; Farivar et al., 2019), reduced amblyopic-eye driven cortical activation (Algaze et al., 2002; Goodyear et al., 2002; Goodyear et al., 2000; Liu et al., 2004; Li et al., 2007b), and fragmented spatial frequency representations across early visual cortex (Choi et al., 2001; Lee et al., 2001; Hess et al., 2009). Together, these results establish that the same feature dimensions measured behaviorally are reliably altered at the cortical level in amblyopia, across species and measurement modalities.

Within this framework, behavioral distortion maps can be viewed as functional readouts of disrupted feature-based representations within retinotopically organized visual cortex, rather than as literal reflections of individual cortical maps. This perspective constrains the behavioral–neural relationship to empirically established feature-level disruptions, while avoiding assumptions of direct one-to-one correspondence between behavioral maps and specific cortical feature maps.

Amblyopia originates from abnormal visual experience during development and is widely recognized as a neurodevelopmental disorder (Polat et al., 2004; Chen et al., 2008; Hernández-Andrés et al., 2025; Zhou and Zhou, 2024; Levi and Li, 2009; Polat et al., 2009; Tsaousis et al., 2023). Accordingly, behavioral interventions such as patching and perceptual learning have demonstrated effectiveness in improving visual function, presumably through their effects on neural processing (Polat et al., 2004; Chen et al., 2008; Hernández-Andrés et al., 2025; Zhou and Zhou, 2024; Levi and Li, 2009; Polat et al., 2009; Tsaousis et al., 2023). This close coupling between behavioral performance and cortical function motivates the use of behavioral mapping approaches as tools for probing neural dysfunction in amblyopia. Comparing behavioral and neural maps may therefore offer a promising strategy for linking perceptual distortions to their underlying neural substrates (Yacoub et al., 2007; Yacoub et al., 2008; Broderick et al., 2022; Farivar et al., 2017; Roth et al., 2022; Goodyear and Menon, 2001).

Importantly, direct empirical evidence linking behavioral distortion maps to specific cortical maps is currently lacking. Establishing such relationships will require targeted neuroimaging and longitudinal studies designed to measure behavioral distortions and neural representations within the same individuals. The present framework is thus intended to generate testable hypotheses, rather than to propose definitive neural mechanisms.

## 5 Discussion

Perceptual distortion is a common feature of amblyopia, but its manifestation varies substantially across individuals and experimental contexts. Importantly, distortion has been linked to functional visual consequences, including disrupted binocular vision and fusion, highlighting its relevance to everyday visual experience (Piano et al., 2015; Piano et al., 2016; Webber, 2018; Wright, 2003; Zagui, 2019). Together, these findings suggest that perceptual distortion constitutes a distinct and meaningful component of amblyopic vision that warrants direct measurement alongside standard clinical metrics.

Importantly, although positional uncertainty and crowding increase with eccentricity in normal peripheral vision, the peripheral distortions observed in amblyopia typically exceed this expected imprecision and reflect abnormal spatial disruption rather than normal peripheral limitations.

Because distortion measurements depend strongly on stimulus and method, a multi-feature approach is necessary to capture the breadth of amblyopic perceptual changes (Section 2).

A practical next step is to incorporate multi-feature, visual-field–wide distortion measurements alongside standard clinical metrics of amblyopia with the goal of evaluating their sensitivity and reliability in longitudinal and treatment contexts. Such measures may help explain why individuals with similar visual acuity can exhibit markedly different perceptual experiences and binocular function, and may offer a more direct way to characterize the aspects of vision that are most relevant for everyday visual performance. Importantly, distortion mapping could prove informative in longitudinal and interventional contexts, for example by revealing perceptual changes that are not captured by acuity alone. However, the potential clinical utility of these measures—for diagnosis, prognosis, or treatment monitoring—remains to be established and will require targeted validation in well-controlled longitudinal and treatment studies.

## 6 Conclusion

In conclusion, perceptual distortions are a clear and important part of amblyopia that need more focused attention. Mapping perceptual distortions across multiple features and visual-field locations offers a promising framework for characterizing amblyopic vision beyond acuity, but its clinical application will require targeted validation in future studies.

## Data Availability

The original contributions presented in the study are included in the article/supplementary material, further inquiries can be directed to the corresponding author.

## References

[ref1] Ahmad Z. Kelly K. R. Freud E. (2023). Reduced perception-action dissociation in children with amblyopia. Neuropsychologia 191:108738. doi: 10.1016/j.neuropsychologia.2023.108738, 38007150

[ref2] Algaze A. Roberts C. Leguire L. Schmalbrock P. Rogers G. (2002). Functional magnetic resonance imaging as a tool for investigating amblyopia in the human visual cortex: a pilot study. JAAPOS 6, 300–308. doi: 10.1067/mpa.2002.124902 12381989

[ref3] Asare A. O. Maurer D. Wong A. M. Saunders N. Ungar W. J. (2023). Cost-effectiveness of universal school-and community-based vision testing strategies to detect amblyopia in children in Ontario, Canada. JAMA Netw. Open 6:e2249384-e. doi: 10.1001/jamanetworkopen.2022.49384, 36598785 PMC9857467

[ref4] Baker F. H. Grigg P. von Noorden G. K. (1974). Effects of visual deprivation and strabismus on the response of neurons in the visual cortex of the monkey, including studies on the striate and prestriate cortex in the normal animal. Brain Res. 66, 185–208. doi: 10.1016/0006-8993(74)90140-1

[ref5] Barbot A. Xue S. Carrasco M. (2021). Asymmetries in visual acuity around the visual field. J. Vis. 21:2. doi: 10.1167/jov.21.1.2, 33393963 PMC7794272

[ref6] Barnes G. Hess R. Dumoulin S. Achtman R. Pike G. (2001). The cortical deficit in humans with strabismic amblyopia. J. Physiol. 533, 281–297. doi: 10.1111/j.1469-7793.2001.0281b.x, 11351035 PMC2278601

[ref7] Barrett B. T. Bradley A. McGraw P. V. (2004). Understanding the neural basis of amblyopia. Neuroscientist 10, 106–117. doi: 10.1177/1073858403262153 15070485

[ref8] Barrett B. T. Pacey I. E. Bradley A. Thibos L. N. Morrill P. (2003). Nonveridical visual perception in human amblyopia. Invest. Ophthalmol. Vis. Sci. 44, 1555–1567. doi: 10.1167/iovs.02-0515, 12657592

[ref9] Bäumer C. Sireteanu R. (2006). Temporal instability in the perception of strabismic amblyopia. Strabismus 14, 59–64. doi: 10.1080/09273970600700939, 16760109

[ref10] Bedell H. E. Flom M. C. (1981). Monocular spatial distortion in strabismic amblyopia. Invest. Ophthalmol. Vis. Sci. 20, 263–268, 7461929

[ref11] Bedell H. E. Flom M. C. Barbeito R. (1985). Spatial aberrations and acuity in strabismus and amblyopia. Invest. Ophthalmol. Vis. Sci. 26, 909–916. 4008207

[ref12] Berman N. Murphy E. H. (1981). The critical period for alteration in cortical binocularity resulting from divergent and convergent strabismus. Dev. Brain Res. 2, 181–202. doi: 10.1016/0165-3806(81)90031-6 7272776

[ref13] Birch E. E. (2013). Amblyopia and binocular vision. Prog. Retin. Eye Res. 33, 67–84. doi: 10.1016/j.preteyeres.2012.11.001, 23201436 PMC3577063

[ref14] Blakemore C. Vital-Durand F. (1986). Effects of visual deprivation on the development of the monkey's lateral geniculate nucleus. J. Physiol. 380, 493–511. 3112372 10.1113/jphysiol.1986.sp016298 PMC1182950

[ref15] Blasdel G. Campbell D. (2001). Functional retinotopy of monkey visual cortex. J. Neurosci. 21, 8286–8301. doi: 10.1523/jneurosci.21-20-08286.2001, 11588200 PMC6763878

[ref16] Blasdel G. Obermayer K. Kiorpes L. (1995). Organization of ocular dominance and orientation columns in the striate cortex of neonatal macaque monkeys. Vis. Neurosci. 12, 589–603. doi: 10.1017/s0952523800008476, 7654611

[ref17] Blasdel G. G. Salama G. (1986). Voltage-sensitive dyes reveal a modular organization in monkey striate cortex. Nature 321, 579–585. doi: 10.1038/321579a0, 3713842

[ref18] Bonneh Y. S. Sagi D. Polat U. (2007). Spatial and temporal crowding in amblyopia. Vis. Res. 47, 1950–1962. doi: 10.1016/j.visres.2007.02.015, 17502115

[ref19] Broderick W. F. Simoncelli E. P. Winawer J. (2022). Mapping spatial frequency preferences across human primary visual cortex. J. Vis. 22:3. doi: 10.1167/jov.22.4.3, 35266962 PMC8934567

[ref20] Carrasco M. Roberts M. Myers C. Shukla L. (2022). Visual field asymmetries vary between children and adults. Curr. Biol. 32, R509–R510. doi: 10.1016/j.cub.2022.04.052, 35671720 PMC9278050

[ref21] Chadnova E. Reynaud A. Clavagnier S. Hess R. F. (2017). Latent binocular function in amblyopia. Vis. Res. 140, 73–80. doi: 10.1016/j.visres.2017.07.014, 28842260

[ref22] Chang K. Fine I. Boynton G. M. (2025). Improving the reliability and accuracy of population receptive field measures using a logarithmically warped stimulus. J. Vis. 25:5. doi: 10.1167/jov.25.1.5 PMC11702787 39752175

[ref23] Chen P. L. Chen J. T. Fu J. J. Chien K. H. Lu D. W. (2008). A pilot study of anisometropic amblyopia improved in adults and children by perceptual learning: an alternative treatment to patching. Ophthalmic Physiol. Opt. 28, 422–428. doi: 10.1111/j.1475-1313.2008.00588.x, 18761479

[ref24] Chen Y. He Z. Mao Y. Chen H. Zhou J. Hess R. F. (2020). Patching and suppression in amblyopia: one mechanism or two? Front. Neurosci. 13:1364. doi: 10.3389/fnins.2019.01364, 32009874 PMC6974542

[ref25] Chino Y. M. Shansky M. S. Jankowski W. L. Banser F. A. (1983). Effects of rearing kittens with convergent strabismus on development of receptive-field properties in striate cortex neurons. J. Neurophysiol. 50, 265–286. doi: 10.1152/jn.1983.50.1.265 6875648

[ref26] Choi M. Y. Lee K. M. Hwang J. M. Choi D. G. Lee D. S. Park K. H. . (2001). Comparison between anisometropic and strabismic amblyopia using functional magnetic resonance imaging. Br. J. Ophthalmol. 85, 1052–1056. doi: 10.1136/bjo.85.9.1052, 11520755 PMC1724107

[ref27] Chung S. T. Li R. W. Levi D. M. (2006). Identification of contrast-defined letters benefits from perceptual learning in adults with amblyopia. Vis. Res. 46, 3853–3861. doi: 10.1016/j.visres.2006.06.014, 16930666 PMC1852540

[ref28] Clavagnier S. Dumoulin S. O. Hess R. F. (2015). Is the cortical deficit in amblyopia due to reduced cortical magnification, loss of neural resolution, or neural disorganization? J. Neurosci. 35, 14740–14755. doi: 10.1523/jneurosci.1101-15.2015, 26538646 PMC6605231

[ref29] Crawford M. L. J. Harwerth R. S. (2004). Ocular dominance column width and contrast sensitivity in monkeys reared with strabismus or anisometropia. Invest. Ophthalmol. Vis. Sci. 45, 3036–3042. doi: 10.1167/iovs.04-0029, 15326118

[ref30] Dallala R. Wang Y.-Z. Hess R. F. (2010). The global shape detection deficit in strabismic amblyopia: contribution of local orientation and position. Vis. Res. 50, 1612–1617. doi: 10.1016/j.visres.2010.05.023 20510268

[ref31] Demanins R. Hess R. F. Williams C. B. Keeble D. R. (1999b). The orientation discrimination deficit in strabismic amblyopia depends upon stimulus bandwidth. Vis. Res. 39, 4018–4031. doi: 10.1016/s0042-6989(99)00107-8, 10748935

[ref32] Demanins R. Wang Y.-Z. Hess R. F. (1999a). The neural deficit in strabismic amblyopia: sampling considerations. Vis. Res. 39, 3575–3585. doi: 10.1016/s0042-6989(99)00070-x, 10746127

[ref33] Drover J. R. Kean P. G. Courage M. L. Adams R. J. (2008). Prevalence of amblyopia and other vision disorders in young Newfoundland and Labrador children. Can. J. Ophthalmol. 43, 89–94. doi: 10.3129/i07-187, 18204498

[ref34] Duman R. Atilla H. Çatak E. (2018). Characteristics of anisometropic patients with and without strabismus. Turkish J. Ophthalmol. 48, 23–26. doi: 10.4274/tjo.44342, 29576894 PMC5854855

[ref35] Dumoulin S. O. Wandell B. A. (2008). Population receptive field estimates in human visual cortex. NeuroImage 39, 647–660. doi: 10.1016/j.neuroimage.2007.09.034 17977024 PMC3073038

[ref36] Farivar R. Clavagnier S. Hansen B. C. Thompson B. Hess R. F. (2017). Non-uniform phase sensitivity in spatial frequency maps of the human visual cortex. J. Physiol. 595, 1351–1363. doi: 10.1113/jp273206, 27748961 PMC5309370

[ref37] Farivar R. Zhou J. Huang Y. Feng L. Zhou Y. Hess R. F. (2019). Two cortical deficits underlie amblyopia: a multifocal fMRI analysis. NeuroImage 190, 232–241. doi: 10.1016/j.neuroimage.2017.09.045, 28943411

[ref38] Field D. J. Hess R. F. (1996). Uncalibrated distortions vs undersampling. Vis. Res. 36, 2121–2124. doi: 10.1016/0042-6989(95)00265-0, 8776478

[ref39] Fronius M. Sireteanu R. (1989). Monocular geometry is selectively distorted in the central visual field of strabismic amblyopes. Invest. Ophthalmol. Vis. Sci. 30, 2034–2044, 2777521

[ref40] Gaier E. D. Hunter D. G. (2017). Advances in Amblyopia Treatment: Paradigm Shifts and Future Directions. Int. Ophthalmol. Clin. 57, 117–128. doi: 10.1097/IIO.0000000000000184, 28885251

[ref41] Goodyear B. G. Menon R. S. (2001). Brief visual stimulation allows mapping of ocular dominance in visual cortex using fMRI. Hum. Brain Mapp. 14, 210–217. doi: 10.1002/hbm.1053, 11668652 PMC6872098

[ref42] Goodyear B. G. Nicolle D. A. Humphrey G. K. Menon R. S. (2000). BOLD fMRI response of early visual areas to perceived contrast in human amblyopia. J. Neurophysiol. 84, 1907–1913. doi: 10.1152/jn.2000.84.4.1907 11024083

[ref43] Goodyear B. G. Nicolle D. A. Menon R. S. (2002). High resolution fMRI of ocular dominance columns within the visual cortex of human amblyopes. Strabismus 10, 129–136. doi: 10.1076/stra.10.2.129.8140, 12221492

[ref44] Goulet L. Farivar R. (2025). NeuroCSF: an fMRI method to measure contrast sensitivity function in human visual cortex. J. Neurophysiol. 133, 1699–1716. doi: 10.1152/jn.00463.2024 40249874

[ref45] Hendrickson A. Movshon J. Eggers H. Gizzi M. Boothe R. Kiorpes L. (1987). Effects of early unilateral blur on the macaque's visual system. II. Anatomical observations. J. Neurosci. 7, 1327–1339. doi: 10.1523/JNEUROSCI.07-05-01327.1987, 3033169 PMC6568823

[ref46] Hernández-Andrés R. Serrano M. Á. Alacreu-Crespo A. Luque M. J. (2025). Randomised trial of three treatments for amblyopia: Vision therapy and patching, perceptual learning and patching alone. Ophthalmic Physiol. Opt. 45, 31–42. doi: 10.1111/opo.13395, 39396111 PMC11629843

[ref47] Hess R. F. (1982). Developmental sensory impairment: amblyopia or tarachopia? Hum. Neurobiol. 1, 17–29. 7185777

[ref48] Hess R. F. (1996). Is amblyopia an impediment to binocular function? Eye 10, 245–249. doi: 10.1038/eye.1996.53 8776455

[ref49] Hess R. F. (2001). Amblyopia: site unseen. Clin. Exp. Optom. 84, 321–336. doi: 10.1111/j.1444-0938.2001.tb06604.x 12366358

[ref50] Hess R. F. Anderson S. (1993). Motion sensitivity and spatial undersampling in amblyopia. Vis. Res. 33, 881–896. doi: 10.1016/0042-6989(93)90071-4, 8506631

[ref51] Hess R. F. Campbell F. W. Greenhalgh T. (1978). On the nature of the neural abnormality in human amblyopia; neural aberrations and neural sensitivity loss. Pflugers Arch. 377, 201–207. doi: 10.1007/bf00584273, 569815

[ref52] Hess R. F. Field D. (1993). Is the increased spatial uncertainty in the normal periphery due to spatial undersampling or uncalibrated disarray? Vis. Res. 33, 2663–2670. doi: 10.1016/0042-6989(93)90226-m 8296463

[ref53] Hess R. F. Field D. J. (1994). Is the spatial deficit in strabismic amblyopia due to loss of cells or an uncalibrated disarray of cells? Vis. Res. 34, 3397–3406. doi: 10.1016/0042-6989(94)90073-6, 7863622

[ref54] Hess R. Field D. Watt R. (1990). “The puzzle of amblyopia” in Vision: Coding and efficiency. ed. Blakemore C. (Cambridge, MA: Cambridge University Press), 267–280.

[ref55] Hess R. F. Li X. Mansouri B. Thompson B. Hansen B. C. (2009). Selectivity as well as sensitivity loss characterizes the cortical spatial frequency deficit in amblyopia. Hum. Brain Mapp. 30, 4054–4069. doi: 10.1002/hbm.20829, 19507159 PMC6871242

[ref56] Hess R. F. Malin S. A. (2003). Threshold vision in amblyopia: orientation and phase. Invest. Ophthalmol. Vis. Sci. 44, 4762–4771. doi: 10.1167/iovs.03-0259, 14578397

[ref57] Hess R. Mansouri B. Thompson B. (2011). Restoration of binocular vision in amblyopia. Strabismus 19, 110–118. doi: 10.3109/09273972.2011.600418, 21870914

[ref58] Hess R. F. Thompson B. (2015). Amblyopia and the binocular approach to its therapy. Vis. Res. 114, 4–16. doi: 10.1016/j.visres.2015.02.009, 25906685

[ref59] Hess R. F. Thompson B. Baker D. H. (2014). Binocular vision in amblyopia: structure, suppression and plasticity. Ophthalmic Physiol. Opt. 34, 146–162. doi: 10.1111/opo.12123, 24588532

[ref60] Himmelberg M. M. Winawer J. Carrasco M. (2020). Stimulus-dependent contrast sensitivity asymmetries around the visual field. J. Vis. 20:18-. doi: 10.1167/jov.20.9.18, 32986805 PMC7533736

[ref61] Himmelberg M. M. Winawer J. Carrasco M. (2022). Linking individual differences in human primary visual cortex to contrast sensitivity around the visual field. Nat. Commun. 13:3309. doi: 10.1038/s41467-022-31041-9, 35697680 PMC9192713

[ref62] Horton J. C. Hocking D. R. Kiorpes L. (1997). Pattern of ocular dominance columns and cytochrome oxidase activity in a macaque monkey with naturally occurring anisometropic amblyopia. Vis. Neurosci. 14, 681–689. doi: 10.1017/s0952523800012645, 9278997

[ref63] Horton J. C. Stryker M. P. (1993). Amblyopia induced by anisometropia without shrinkage of ocular dominance columns in human striate cortex. Proc. Natl. Acad. Sci. USA 90, 5494–5498. doi: 10.1073/pnas.90.12.5494, 8390668 PMC46747

[ref64] Hou C. Nicholas S. C. (2022). Perceptual learning with dichoptic attention tasks improves attentional modulation in V1 and IPS and reduces interocular suppression in human amblyopia. Sci. Rep. 12:9660. doi: 10.1038/s41598-022-13747-4, 35690626 PMC9188564

[ref65] Hozumi K. Yagasaki T. Yokoyama Y. Yagasaki A. Haga Y. Eboshita R. (2023). Relationship between suppression scotomas and stereoacuity in anisometropic amblyopia with successfully treated visual acuity. Invest. Ophthalmol. Vis. Sci. 64:16. doi: 10.1167/iovs.64.11.16, 37561448 PMC10424799

[ref66] Hubel D. H. Wiesel T. N. (1998). Early exploration of the visual cortex. Neuron 20, 401–412. 9539118 10.1016/s0896-6273(00)80984-8

[ref67] Hubeland T. D. Wiesel N. (1977). Functionalarchitectureofmacaquemonkeyvisual cortex. Proc Roy Soc Lond B. 198, 1–59.20635

[ref68] Hussain Z. McGraw P. V. (2022). Disruption of Positional Encoding at Small Separations in the Amblyopic Periphery. Invest. Ophthalmol. Vis. Sci. 63:15. doi: 10.1167/iovs.63.4.15, 35446345 PMC9034712

[ref69] Hussain Z. Svensson C.-M. Besle J. Webb B. S. Barrett B. T. McGraw P. V. (2015). Estimation of cortical magnification from positional error in normally sighted and amblyopic subjects. J. Vis. 15:25. doi: 10.1167/15.2.25, 25761341 PMC4341865

[ref70] Iftime A. Bäumer C. C. Sireteanu R. (2007). An automated method for creating simulated distorted images in amblyopic vision. Strabismus 15, 21–27. doi: 10.1080/09273970601175016 17523042

[ref71] Jigo M. Tavdy D. Himmelberg M. M. Carrasco M. (2023). Cortical magnification eliminates differences in contrast sensitivity across but not around the visual field. eLife 12:e84205. doi: 10.7554/eLife.84205, 36961485 PMC10089656

[ref72] Kalpadakis-Smith A. V. Tailor V. K. Dahlmann-Noor A. H. Greenwood J. A. (2022). Crowding changes appearance systematically in peripheral, amblyopic, and developing vision. J. Vis. 22:3. doi: 10.1167/jov.22.6.3 PMC9078053 35506917

[ref73] Kim D.-S. Matsuda Y. Ohki K. Ajima A. Tanaka S. (1999). Geometrical and topological relationships between multiple functional maps in cat primary visual cortex. Neuroreport 10, 2515–2522. doi: 10.1097/00001756-199908200-00015, 10574362

[ref74] Kiorpes L. Kiper D. C. O'Keefe L. P. Cavanaugh J. R. Movshon J. A. (1998). Neuronal correlates of amblyopia in the visual cortex of macaque monkeys with experimental strabismus and anisometropia. J. Neurosci. 18, 6411–6424. doi: 10.1523/JNEUROSCI.18-16-06411.1998, 9698332 PMC6793177

[ref75] Kirsch W. Kunde W. (2023). Human perception of spatial frequency varies with stimulus orientation and location in the visual field. Sci. Rep. 13:17656. doi: 10.1038/s41598-023-44673-8, 37848541 PMC10582250

[ref76] Kwon M. Wiecek E. Dakin S. C. Bex P. J. (2015). Spatial-frequency dependent binocular imbalance in amblyopia. Sci. Rep. 5:17181. doi: 10.1038/srep17181, 26603125 PMC4658600

[ref77] Lagreze W.-D. Sireteanu R. (1991). Two-dimensional spatial distortions in human strabismic amblyopia. Vis. Res. 31, 1271–1288. 1891818 10.1016/0042-6989(91)90051-6

[ref78] Lee K.-M. Lee S.-H. Kim N.-Y. Kim C.-Y. Sohn J.-W. Choi M. Y. . (2001). Binocularity and spatial frequency dependence of calcarine activation in two types of amblyopia. Neurosci. Res. 40, 147–153. doi: 10.1016/s0168-0102(01)00220-6, 11377753

[ref79] LeVay S. Connolly M. Houde J. Van Essen D. C. (1985). The complete pattern of ocular dominance stripes in the striate cortex and visual field of the macaque monkey. J. Neurosci. 5, 486–501. doi: 10.1523/JNEUROSCI.05-02-00486.1985, 3973679 PMC6565187

[ref80] Levi D. M. (2013). Linking assumptions in amblyopia. Vis. Neurosci. 30, 277–287. doi: 10.1017/s0952523813000023, 23879956 PMC5533593

[ref81] Levi D. M. Li R. W. (2009). Perceptual learning as a potential treatment for amblyopia: a mini-review. Vis. Res. 49, 2535–2549. doi: 10.1016/j.visres.2009.02.010, 19250947 PMC2764839

[ref82] Li X. Dumoulin S. O. Mansouri B. Hess R. F. (2007a). The fidelity of the cortical retinotopic map in human amblyopia. Eur. J. Neurosci. 25, 1265–1277. doi: 10.1111/j.1460-9568.2007.05356.x, 17425555

[ref83] Li X. Dumoulin S. O. Mansouri B. Hess R. F. (2007b). Cortical deficits in human amblyopia: their regional distribution and their relationship to the contrast detection deficit. Invest. Ophthalmol. Vis. Sci. 48, 1575–1591. doi: 10.1167/iovs.06-1021 17389487

[ref84] Li R. W. Klein S. A. Levi D. M. (2008). Prolonged perceptual learning of positional acuity in adult amblyopia: perceptual template retuning dynamics. J. Neurosci. 28, 14223–14229. doi: 10.1523/jneurosci.4271-08.2008, 19109504 PMC2765479

[ref85] Li J. Li J. Chen Z. Liu J. Yuan J. Cai X. . (2017). Spatial and global sensory suppression mapping encompassing the central 10 field in anisometropic amblyopia. Invest. Ophthalmol. Vis. Sci. 58, 481–491. doi: 10.1167/iovs.16-20298, 28122090

[ref86] Liu G. T. Miki A. Francis E. Quinn G. E. Modestino E. J. Bonhomme G. R. . (2004). Eye dominance in visual cortex in amblyopia using functional magnetic resonance imaging. J. AAPOS 8, 184–186. doi: 10.1016/j.jaapos.2003.11.001 15088055

[ref87] Lv Z. Tao Z. Hu G. Deng H. (2024). Significance of binocular fusion in enhancing visual acuity during amblyopia treatment. Transl. Pediatr. 13, 1767–1776. doi: 10.21037/tp-24-125, 39524389 PMC11543132

[ref88] Lygo F. A. (2019). Neuroimaging of binocular vision in human amblyopia. New York, NY: University of York.

[ref89] Mansouri B. Hansen B. C. Hess R. F. (2009). Disrupted retinotopic maps in amblyopia. Invest. Ophthalmol. Vis. Sci. 50, 3218–3225. doi: 10.1167/iovs.08-2914, 19255157

[ref90] Mansouri B. Hess R. F. Allen H. A. (2007). Orientation variance discrimination in amblyopia. J. Opt. Soc. Am. A Opt. Image Sci. Vis. 24, 2499–2504. doi: 10.1364/josaa.24.002499, 17767220

[ref91] Mao Y. Min S. H. Chen S. Gong L. Chen H. Hess R. F. . (2020). Binocular imbalance in amblyopia depends on spatial frequency in binocular combination. Invest. Ophthalmol. Vis. Sci. 61:7-. doi: 10.1167/iovs.61.8.7, 32634205 PMC7425706

[ref92] Mathew J. A. Shah S. A. Simon J. W. (2011). Varying difficulty of Snellen letters and common errors in amblyopic and fellow eyes. Arch. Ophthalmol. 129, 184–187. doi: 10.1001/archophthalmol.2010.369, 21320964

[ref93] McConaghy J. R. McGuirk R. (2019). Amblyopia: detection and treatment. Am. Fam. Physician 100, 745–750. 31845774

[ref94] McKee S. P. Levi D. M. Movshon J. A. (2003). The pattern of visual deficits in amblyopia. J. Vis. 3:5. doi: 10.1167/3.5.5 12875634

[ref95] Molaei H. Farishta R. A. Farivar R. (2025a). Multi-feature mapping of distortions in amblyopia with localized sampling. Invest. Ophthalmol. Vis. Sci. 66:37. doi: 10.1167/iovs.66.6.37, 40492986 PMC12165260

[ref96] Molaei H. Farishta R. A. Farivar R. (2025b). Letter distortion mapping in amblyopia: spatial patterns, stability, and relationship to visual acuity. Invest. Ophthalmol. Vis. Sci. 66:65. doi: 10.1167/iovs.66.15.65, 41533934 PMC12742594

[ref97] Morsi A. Y. Goffaux V. Greenwood J. A. (2024). The resolution of face perception varies systematically across the visual field. PLoS One 19:e0303400. doi: 10.1371/journal.pone.0303400, 38739635 PMC11090322

[ref98] Mostafaie A. Ghojazadeh M. Hosseinifard H. Manaflouyan H. Farhadi F. Taheri N. . (2020). A systematic review of Amblyopia prevalence among the children of the world. Rom. J. Ophthalmol. 64, 342–355. doi: 10.22336/rjo.2020.56, 33367172 PMC7739017

[ref99] Movshon J. A. Eggers H. M. Gizzi M. S. Hendrickson A. E. Kiorpes L. Boothe R. G. (1987). Effects of early unilateral blur on the macaque's visual system. III. Physiological observations. J. Neurosci. 7, 1340–1351. 3572484 10.1523/JNEUROSCI.07-05-01340.1987 PMC6568830

[ref100] Movshon J. A. Thompson I. Tolhurst D. (1978). Spatial and temporal contrast sensitivity of neurones in areas 17 and 18 of the cat's visual cortex. J. Physiol. 283, 101–120. doi: 10.1113/jphysiol.1978.sp012490, 722570 PMC1282767

[ref101] Naheed F. Ullah S. Asgher M. Qayyum S. (2024). Comparison of contrast sensitivity among strabismic and anisometropic amblyopes and its association with disease-related parameters. Saudi J. Ophthalmol. 38, 83–88. doi: 10.4103/sjopt.sjopt_7_23, 38628417 PMC11017012

[ref102] Nasr S. Skerswetat J. Gaier E. D. Malladi S. N. Kennedy B. Tootell R. B. . (2024). Using high-resolution functional MRI to differentiate impacts of strabismic and anisometropic amblyopia on evoked ocular dominance activity in humans. bioRxiv. doi: 10.1101/2024.02.11.579855

[ref103] Nauhaus I. Nielsen K. J. Disney A. A. Callaway E. M. (2012). Orthogonal micro-organization of orientation and spatial frequency in primate primary visual cortex. Nat. Neurosci. 15, 1683–1690. doi: 10.1038/nn.3255, 23143516 PMC3509274

[ref104] Obermayer K. Blasdel G. G. (1993). Geometry of orientation and ocular dominance columns in monkey striate cortex. J. Neurosci. 13, 4114–4129. doi: 10.1523/JNEUROSCI.13-10-04114.1993, 8410181 PMC6576395

[ref105] O'Connor A. R. Birch E. E. Anderson S. Draper H. (2010). Relationship between binocular vision, visual acuity, and fine motor skills. Optom. Vis. Sci. 87, 942–947. doi: 10.1097/OPX.0b013e3181fd132e, 21057348

[ref106] Pardhan S. Whitaker A. (2000). Binocular summation in the fovea and peripheral field of anisometropic amblyopes. Curr. Eye Res. 20, 35–44. doi: 10.1076/0271-3683(200001)2011-hft035 10611713

[ref107] Piano M. E. Bex P. J. Simmers A. J. (2015). Perceptual Visual Distortions in Adult Amblyopia and Their Relationship to Clinical Features. Invest. Ophthalmol. Vis. Sci. 56, 5533–5542. doi: 10.1167/iovs.15-17071, 26284559 PMC4544188

[ref108] Piano M. E. Bex P. J. Simmers A. J. (2016). Perceived Visual Distortions in Juvenile Amblyopes During/Following Routine Amblyopia Treatment. Invest. Ophthalmol. Vis. Sci. 57, 4045–4054. doi: 10.1167/iovs.16-19210, 27494346

[ref109] Polat U. Ma-Naim T. Belkin M. Sagi D. (2004). Improving vision in adult amblyopia by perceptual learning. Proc. Natl. Acad. Sci. USA 101, 6692–6697. doi: 10.1073/pnas.0401200101, 15096608 PMC404107

[ref110] Polat U. Ma-Naim T. Spierer A. (2009). Treatment of children with amblyopia by perceptual learning. Vis. Res. 49, 2599–2603. doi: 10.1016/j.visres.2009.07.008, 19622368

[ref111] Pons C. Jin J. Mazade R. Dul M. Zaidi Q. Alonso J.-M. (2019). Amblyopia affects the ON visual pathway more than the OFF. J. Neurosci. 39, 6276–6290. doi: 10.1523/JNEUROSCI.3215-18.2019, 31189574 PMC6687897

[ref112] Pugh M. (1958). Visual distortion in amblyopia. Br. J. Ophthalmol. 42:449. doi: 10.1136/bjo.42.8.449, 13572757 PMC509680

[ref113] Pugh M. (1962). Amblyopia and the retina. Br. J. Ophthalmol. 46:193. doi: 10.1136/bjo.46.4.193, 18170771 PMC510186

[ref114] Rentschler I. Hilz R. (1979). Abnormal orientation selectivity in both eyes of strabismic amblyopes. Exp. Brain Res. 37, 187–191. doi: 10.1007/bf01474265 488215

[ref115] Rohr J. T. D. Isaac C. R. de Lima A. A. Garcia A. Dos Santos P. M. Tavares M. C. H. (2022). Study of Geometric Illusory Visual Perception–A New Perspective in the Functional Evaluation of Children With Strabismus. Front. Hum. Neurosci. 16:769412. doi: 10.3389/fnhum.2022.769412 35496072 PMC9043129

[ref116] Roth Z. N. Kay K. Merriam E. P. (2022). Natural scene sampling reveals reliable coarse-scale orientation tuning in human V1. Nat. Commun. 13:6469. doi: 10.1038/s41467-022-34134-7, 36309512 PMC9617970

[ref117] Roux-Sibilon A. Peyrin C. Greenwood J. A. Goffaux V. (2023). Radial bias in face identification. Proc. R. Soc. Lond. B Biol. Sci. 290:20231118. doi: 10.1098/rspb.2023.1118, 37357864 PMC10291718

[ref118] Sharma V. Levi D. M. Coletta N. J. (1999). Sparse-sampling of gratings in the visual cortex of strabismic amblyopes. Vis. Res. 39, 3526–3536. doi: 10.1016/s0042-6989(99)00028-0, 10746124

[ref119] Shiraishi Y. Wakayama A. Matsumoto F. Tanabe F. Kusaka S. (2023). The association between improvement of stereoacuity and suppression in the treatment of anisometropic amblyopia. Clin. Ophthalmol. 17, 1545–1553. doi: 10.2147/opth.s412194, 37284056 PMC10241209

[ref120] Shooner C. Hallum L. E. Kumbhani R. D. Ziemba C. M. Garcia-Marin V. Kelly J. G. . (2015). Population representation of visual information in areas V1 and V2 of amblyopic macaques. Vis. Res. 114, 56–67. doi: 10.1016/j.visres.2015.01.012, 25637856 PMC4519437

[ref121] Simmers A. J. Bex P. J. (2004). The representation of global spatial structure in amblyopia. Vis. Res. 44, 523–533. doi: 10.1016/j.visres.2003.10.010 14680777

[ref122] Sireteanu R. Bäumer C. C. Iftime A. (2008). Temporal instability in amblyopic vision: relationship to a displacement map of visual space. Invest. Ophthalmol. Vis. Sci. 49, 3940–3954. doi: 10.1167/iovs.07-0351, 18765634

[ref123] Sireteanu R. Bäumer C. C. Sârbu C. Iftime A. (2007). Spatial and temporal misperceptions in amblyopic vision. Strabismus 15, 45–54. doi: 10.1080/09273970601180263, 17523046

[ref124] Sireteanu R. Lagreze W. D. Constantinescu D. H. (1993). Distortions in two-dimensional visual space perception in strabismic observers. Vis. Res. 33, 677–690. doi: 10.1016/0042-6989(93)90188-3, 8351840

[ref125] Sireteanu R. Thiel A. Fikus S. Iftime A. (2008). Patterns of spatial distortions in human amblyopia are invariant to stimulus duration and instruction modality. Vis. Res. 48, 1150–1163. doi: 10.1016/j.visres.2008.01.028, 18343480

[ref126] Skottun B. Bradley A. Freeman R. (1986). Orientation discrimination in amblyopia. Invest. Ophthalmol. Vis. Sci. 27, 532–537. 3957571

[ref127] Smith E. L. Chino Y. M. Ni J. Cheng H. Crawford M. L. Harwerth R. S. (1997). Residual binocular interactions in the striate cortex of monkeys reared with abnormal binocular vision. J. Neurophysiol. 78, 1353–1362. doi: 10.1152/jn.1997.78.3.1353, 9310426

[ref128] Swindale N. V. Mitchell D. E. (1994). Comparison of receptive field properties of neurons in area 17 of normal and bilaterally amblyopic cats. Exp. Brain Res. 99, 399–410. doi: 10.1007/bf00228976, 7957719

[ref129] Szinte M. Lascombes U. Sheynin Y. Levi D. M. Silver M. A. Chopin A. (2024). Modeling retinotopic maps in amblyopia reveals cortical reorganization across the visual hierarchy. J. Vis. 24:551. doi: 10.1167/jov.24.10.551

[ref130] Thibos L. N. Bradley A. (1993). New methods for discriminating neural and optical losses of vision. Optom. Vis. Sci. 70, 279–287. doi: 10.1097/00006324-199304000-00006, 8502456

[ref131] Thibos L. N. Bradley A. (1995). “Modeling off-axis vision II: the effect of spatial filtering and sampling by retinal neurons” in Vision Models For Target Detection And Recognition: In Memory of Arthur Menendez. ed. Menendez A. R. (London: World Scientific), 338–379.

[ref132] Tootell R. B. Switkes E. Silverman M. S. Hamilton S. L. (1988). Functional anatomy of macaque striate cortex. II. Retinotopic organization. J. Neurosci. 8, 1531–1568. doi: 10.1523/JNEUROSCI.08-05-01531.1988, 3367210 PMC6569212

[ref133] Tsai L.-T. Liao K.-M. Hou C.-H. Jang Y. Chen C.-C. (2024). Visual field asymmetries in visual word form identification. Vis. Res. 220:108413. doi: 10.1016/j.visres.2024.108413, 38613969

[ref134] Tsaousis K. T. Mousteris G. Diakonis V. Chaloulis S. (2023). Current developments in the management of amblyopia with the use of perceptual learning techniques. Medicina (Kaunas) 60:48. doi: 10.3390/medicina60010048, 38256309 PMC10821148

[ref135] Vandenbussche E. Vogels R. Orban G. A. (1986). Human orientation discrimination: changes with eccentricity in normal and amblyopic vision. Invest. Ophthalmol. Vis. Sci. 27, 237–245. 3943947

[ref136] Wallace D. K. Repka M. X. Lee K. A. Melia M. Christiansen S. P. Morse C. L. . (2018). Amblyopia preferred practice pattern®. Ophthalmology 125, P105–P142. doi: 10.1016/j.ophtha.2022.11.003 29108744

[ref137] Wang Y. Wu Y. Luo L. Li F. (2023). Structural and functional alterations in the brains of patients with anisometropic and strabismic amblyopia: a systematic review of magnetic resonance imaging studies. Neural Regen. Res. 18, 2348–2356. doi: 10.4103/1673-5374.371349, 37282452 PMC10360096

[ref138] Webber A. L. (2018). The functional impact of amblyopia. Clin. Exp. Optom. 101, 443–450. doi: 10.1111/cxo.12663, 29484704

[ref139] Webber A. L. Schmid K. L. Baldwin A. S. Hess R. F. (2020). Suppression rather than visual acuity loss limits stereoacuity in amblyopia. Invest. Ophthalmol. Vis. Sci. 61:50. doi: 10.1167/iovs.61.6.50 PMC7419721 32579677

[ref140] Wiecek E. Kosovicheva A. Ahmed Z. Nabasaliza A. Kazlas M. Chan K. . (2024). Peripheral binocular imbalance in anisometropic and strabismic amblyopia. Invest. Ophthalmol. Vis. Sci. 65:36. doi: 10.1167/iovs.65.4.36, 38652649 PMC11044833

[ref141] Wiecek E. Ramirez L. D. Klimova M. Ling S. (2024). Are visual deficits in amblyopia driven by spatial frequency tuning? J. Vis. 24:913-. doi: 10.1167/jov.24.10.913

[ref142] Williams D. R. (1985). Aliasing in human foveal vision. Vis. Res. 25, 195–205. doi: 10.1016/0042-6989(85)90113-0, 4013088

[ref143] Wright K. W. (2003). Visual development and amblyopia. Pediatric ophthalmology and strabismus. Cham: Springer, 157–171.

[ref144] Xiao O. Morgan I. G. Ellwein L. B. He M. Group RESiCS (2015). Prevalence of amblyopia in school-aged children and variations by age, gender, and ethnicity in a multi-country refractive error study. Ophthalmology 122, 1924–1931. doi: 10.1016/j.ophtha.2015.05.034, 26278861 PMC6029943

[ref145] Yacoub E. Harel N. Uğurbil K. (2008). High-field fMRI unveils orientation columns in humans. Proc. Natl. Acad. Sci. USA 105, 10607–10612. doi: 10.1073/pnas.0804110105, 18641121 PMC2492463

[ref146] Yacoub E. Shmuel A. Logothetis N. Uğurbil K. (2007). Robust detection of ocular dominance columns in humans using Hahn Spin Echo BOLD functional MRI at 7 Tesla. NeuroImage 37, 1161–1177. doi: 10.1016/j.neuroimage.2007.05.020, 17702606 PMC2040323

[ref147] Yap T. P. Luu C. D. Suttle C. Chia A. Boon M. Y. (2020). Effect of stimulus orientation on visual function in children with refractive amblyopia. Invest. Ophthalmol. Vis. Sci. 61:5. doi: 10.1167/iovs.61.5.5 PMC7405838 32392311

[ref148] Yinon U. (1975). Eye rotation in developing kittens: the effect on ocular dominance and receptive field organization of cortical cells. Exp. Brain Res. 24, 215–218. doi: 10.1007/bf00234065, 1218553

[ref149] Yinon U. (1976). Eye rotation surgically induced in cats modifies properties of cortical neurons. Exp. Neurol. 51, 603–627. doi: 10.1016/0014-4886(76)90184-9, 1278283

[ref150] Yu H. Farley B. J. Jin D. Z. Sur M. (2005). The coordinated mapping of visual space and response features in visual cortex. Neuron 47, 267–280. doi: 10.1016/j.neuron.2005.06.011, 16039568

[ref151] Zagui R. B. (2019). Amblyopia: types, diagnosis, treatment, and new perspectives. London: American Academy of Ophthalmology, 2–4.

[ref152] Zhao W. Jia W.-L. Chen G. Luo Y. Lin B. He Q. . (2017). A complete investigation of monocular and binocular functions in clinically treated amblyopia. Sci. Rep. 7:10682. doi: 10.1038/s41598-017-11124-0, 28878318 PMC5587672

[ref153] Zhou J. Li L. Zhang P. Xi J. Zhou Y. Lu Z.-L. . (2015). Tilt after-effect from high spatial-frequency patterns in the amblyopic eye of adults with anisometropic amblyopia. Sci. Rep. 5:8728. doi: 10.1038/srep08728 25735899 PMC4348659

[ref154] Zhou S. Zhou J. (2024). New advances in amblyopia therapy: early patching is more effective than extended optical treatment. Lancet 403, 1725–1727. doi: 10.1016/s0140-6736(24)00350-7, 38704157

[ref155] Zhu X. R. Hess R. F. Baldwin A. S. (2024). Human visual performance for identifying letters affected by physiologically-inspired scrambling. bioRxiv 27:583720.

